# Long term dental implant survival and bone level changes with special emphasis on radiation therapy after free fibula flap reconstruction – a retrospective study

**DOI:** 10.1007/s00784-025-06587-9

**Published:** 2025-11-10

**Authors:** Jonas Wüster, Lisa Mahlow, Norbert Neckel, Carsten Rendenbach, Max Heiland, Kirstin Vach, Claudia Sachse, Katja Nelson, Florian Kernen, Susanne Nahles

**Affiliations:** 1 Department of Oral and Maxillofacial Surgery, Faculty of Medicine, University Medical Center Freiburg, Albert Ludwig University of Freiburg, Freiburg, Baden-Württemberg Germany; 2 Department of Oral and Maxillofacial Surgery, Charité—Universitätsmedizin Berlin, Corporate Member of Freie Universität Berlin, and Humboldt-Universität zu Berlin, Berlin, Germany; 3 Institute of Medical Biometry and Statistics, Faculty of Medicine and Medical Center, University of Freiburg, Stefan-Meier-Straße 26, Freiburg, 79106 Germany

**Keywords:** Oral cancer, Prosthodontic rehabilitation, Dental implants, Irradiated patients, Dental implant success, Dental implant survival

## Abstract

**Objectives:**

This study aimed to investigate the long-term survival of dental implants in patients after ablative resection of the maxilla or mandible and consecutive reconstruction with microvascular free fibula flaps (FFF).

**Materials and methods:**

Patients treated with a FFF reconstruction of the mandible/maxilla and dental implant insertion were included. Implant survival and the influence of age, gender, radiotherapy, and localization were evaluated. Moreover, mesial and distal bone resorption was recorded. Implant survival was assessed using Kaplan–Meier methods and Cox regression, while bone loss was analyzed with linear mixed models. Multiple testing was adjusted by Scheffé’s method; significance was set at *p* < 0.05.

**Results:**

In total, 53 patients with 257 implants were evaluated. The mean observation period was 64 months. The cumulative survival rate was 98.4% at 1 year, 95.7% after 3 years, and 91.8% at 5 years. After five years, adjuvant radiotherapy was associated with reduced implant survival (73.8%) and significantly increased distal bone loss (*p* = 0.009). Moreover, native bone (mesial: 3.2 ± 0.6; distal 3.2 ± 0.8) demonstrated significantly greater bone loss compared with fibula bone (mesial: 2.6 ± 1.6; distal 2.8 ± 1.7).

**Conclusion:**

Adjuvant radiotherapy results in a markedly reduced implant survival and a significantly greater marginal bone (distal) loss after five years.

**Clinical relevance:**

Dental implants reveal higher survival in fibula bone after mandibular/maxillary reconstruction with microvascular FFF when irradiation was performed prior to reconstruction with a FFF and in patients without irradiation.

**Supplementary Information:**

The online version contains supplementary material available at 10.1007/s00784-025-06587-9.

## Introduction

Head and neck squamous cell carcinoma (HNSCC) ranks as the 7th most common cancer worldwide according to GLOBOCAN, accounting for approximately 4.5% of cancer diagnoses [1, 2]. Within this heterogeneous group, oral (squamous cell) cancer is the most common tumor, which is often treated with multimodal therapy, including ablative surgery and, if required, adjuvant radiation or radiochemotherapy [3]. However, there are also benign tumors in the head and neck region that require surgical treatment and reconstructive measures [4]. If the tumor is in direct proximity to the mandible, a segmental mandibulectomy is typically required [5], resulting in significant deficits in both soft and hard tissues. This can lead to disrupted myodynamics and malocclusion, thereby affecting the patient’s quality of life [6–8], which also applies to the maxilla. For such critically sized bone defects, the microvascular free fibula flap (FFF) remains the “gold standard” in osseous reconstruction [9] due to its sufficient bone quantity, ideal contouring capabilities, vascular supply, and long pedicle [10, 11]. Recently, the integration of computer-aided design and computer-assisted manufacturing (CAD/CAM) templates and plates in reconstructive head and neck surgery has revolutionized osseous reconstruction with FFFs [12, 13]. This advancement has resulted in highly accurate reconstructions, reduced surgery time, and improved aesthetic and functional outcomes [14, 15]. Despite these advances, prosthodontic rehabilitation in these patients remains challenging, as conventional prosthodontic options are often limited [16]. However, the FFF offers a significant advantage by facilitating dental implant placement and subsequent prosthodontic rehabilitation [17–19]. Nevertheless, inconsistent and often low rates of prosthesis-based oral rehabilitation following FFF reconstruction remain a concern [20–22]. Additionally, dental implant survival rates in patients who have undergone jaw reconstruction using FFFs vary widely, ranging from 78% to 96%, with the impact of irradiation being a subject of ongoing debate [20, 23, 24]. Moreover, these studies often lack long-term follow-up [25], evaluation of mesial and distal peri-implant bone levels, an adequate sample size of included patients [26], information on the prosthodontic reconstruction used, or consideration of the effects of radiotherapy [27]. It is known that patients who have undergone radiotherapy for head and neck cancers often suffer from compromised bone vascularity, reduced cellular activity, and increased risk of complications such as osteoradionecrosis (ORN), which can adversely affect implant survival and long-term success. Radiation-induced changes in bone physiology can lead to impaired osseointegration, a critical factor for implant stability. Furthermore, the risk of postoperative infection and implant failure increases in irradiated tissues. Literature regarding patients exposed to therapeutic radiation shows lower dental implant survival rates (72%−84.6%) compared to non-irradiated patients (95%−95.3%) in native bone [28, 29].

Therefore, our study aimed to evaluate the long-term survival of dental implants and associated potential clinical parameters in patients who underwent ablative tumor surgery followed by reconstruction with an FFF. Additionally, we analyzed the impact of irradiation on the survival rate and peri-implant bone level changes with consideration of gender.

## Methods

### Ethical statement

This study was approved by the Institutional Review Board of the Faculty of Medicine Charité Berlin (EA4/064/18) and was performed in accordance with the Helsinki Declaration of 1964, as revised in 2013.

### Patient consent statement

In accordance with the statement of the ethics committee, informed consent was obtained from all participants included in this study.

### Inclusion and exclusion criteria

Patients treated from 2018 to 2024 at the Department of Oral and Maxillofacial Surgery at Charité—Universitätsmedizin Berlin with ablative surgery of the mandible or maxilla, followed by reconstruction using a microvascular FFF, were eligible for inclusion. Patients with significantly compromised general health, such as those with immunocompromised conditions (e.g. autoimmune diseases, HIV infection, or undergoing cortisone treatment), were excluded from the study.

### Patients

**Fig. 1:**
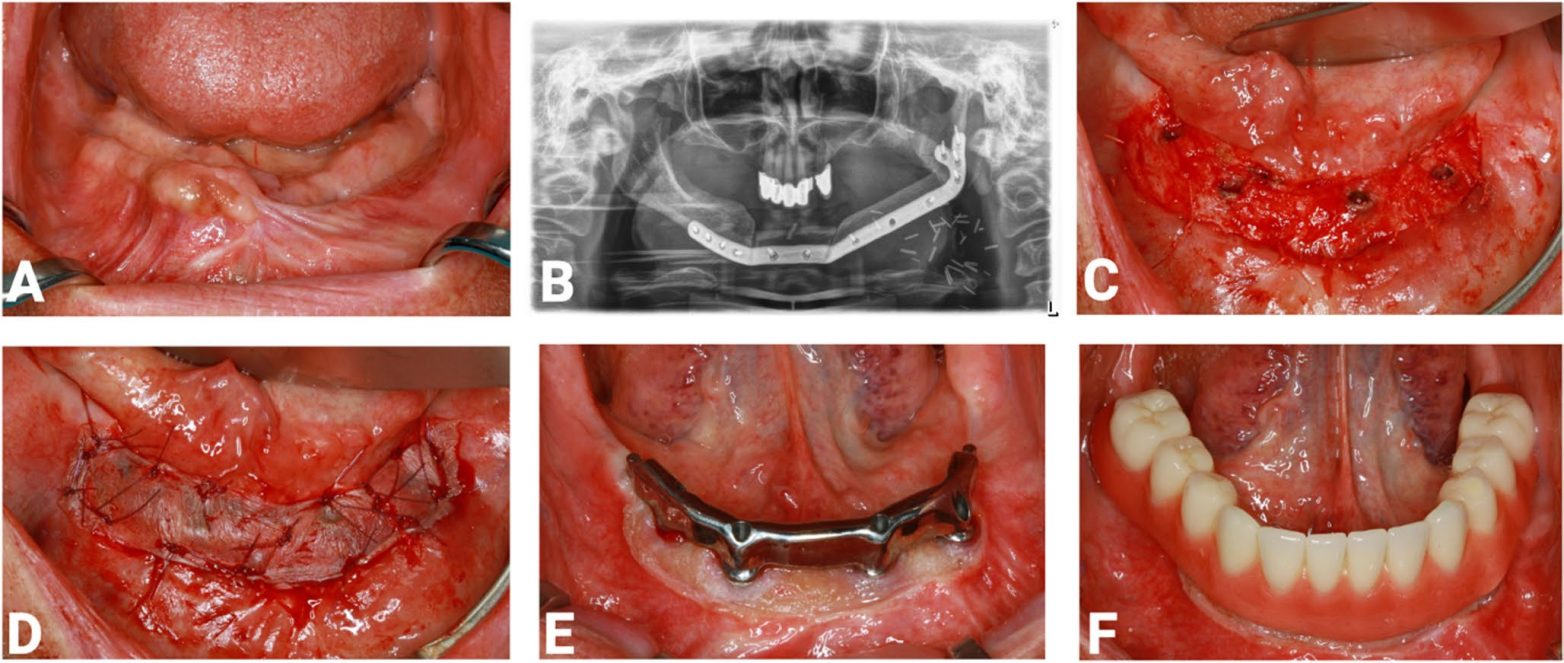
**A** Intraoral situation after reconstruction of the mandible using a free microvascular fibula flap (FFF). **B** Postoperative orthopantomogram with reconstruction plate in place. **C** Preparation of the mucosal flap with further lingual and buccal advancement. **D** Vestibuloplasty with placement of a 0.2 mm split-thickness skin graft, sutured in position. **E** Situation after vestibuloplasty with implant-supported bar. **F** Final situation with removable bar-supported overdentures

Some patients received not only surgical treatment but also additional radiation/radiochemotherapy. In such cases, dental implant placement was performed after a minimum of 6 months. In all patients, a vestibuloplasty (VP) with a split-thickness skin graft was performed as routine treatment in the reconstructed jaw (Fig. 1), following the method described by Heberer and Nelson [16]. Surgical resection of the tumor, dental implant placement, soft tissue modification procedures, prosthodontic treatment with implant-supported prostheses, and regular follow-up examinations were conducted at the Department of Oral and Maxillofacial Surgery, Charité—Universitätsmedizin Berlin. Additionally, different types of prosthodontic restorations were evaluated for the respective jaws, both clinically and using x-ray (orthopantomogram; device: Planmeca ProMax 3DMax, Pro Face Med Series H23 12 kV).

### Surgical treatment

Implant placement was performed as described in previous studies [19]. Additionally, the respective manufacturers’ protocols for Camlog Screw-Line in the lower jaw and Camlog Progressive line in the upper jaw (Camlog Biotechnologies AG, Basel, Switzerland) or ICX Premium in the mandible and ICX Active in the maxilla (Medentis Medical GmbH, Bad Neuenahr-Ahrweiler, Germany) were followed. Using local anesthesia (Ultracain D-S forte 1:100 000), dental implants were placed epicrestally. The subsequent healing period was 2 months for the mandible and 3 months for the maxilla, with no distinction made between reconstructed and non-reconstructed bone as well as between irradiated and non-irradiated jaws. Starting 1 day pre-operatively, patients were prescribed an oral antibiotic regimen of amoxicillin/clavulanic acid (875/125 mg every 12 h), which continued for 4 days post-operatively. For patients with penicillin intolerance, clindamycin (300 mg, 3 times per day for 5 days) and, after 2021, an azithromycin 3-day dose pack were administered.

### Clinical and radiological evaluation

After dental implant placement and the completion of prosthodontic therapy, regular clinical follow-ups were performed as previously described. Early dental implant failure was defined as a dental implant loss that occurred within 12 months after implant placement. Panoramic radiographs (orthopantomograms) were taken following dental rehabilitation using the Planmeca ProMax 3DMax (Pro Face Med Series H23 12 kV) in a standardized setting. The dentition of the opposing arch was categorized as fully dentate, partially edentulous, completely edentulous, or restored with dental implants. The reconstructed defects were classified according to the systems of [30] for the mandible and [31] for the maxilla. Follow-ups were conducted every 3 months during the first year and then annually thereafter. Clinical examinations followed a standardized protocol, including radiographic assessments at 1, 2, 3, and 5 years using the same imaging device. Peri-implant bone level changes were evaluated based on the method described by Gomez-Roman et al., analyzing mesial and distal peri-implant regions [32]. Additionally, patients underwent professional dental prophylaxis every 3 months, performed by trained personnel.

### Statistics

Statistical analyses were performed using STATA (Version 19.0, College Station, TX, USA) For descriptive analysis means, standard deviations were calculated and boxplots used for visualization Implant survival probabilities were estimated using the Kaplan–Meier method, and group comparisons were conducted using cox regression with adjustment for age, gender, implant length and implant diameter under consideration of the clustering of the observations. For comparison of bone loss values between subgroups within one timepoint linear mixed models with patient as random factor and adjustment for gender age and jaw were used. For all subsequent pairwise comparisons the method of Scheffe’ was used to correct for multiple testing. A significance level of *p* = 0.05 was applied for all statistical tests.

## Results

**Fig. 2:**
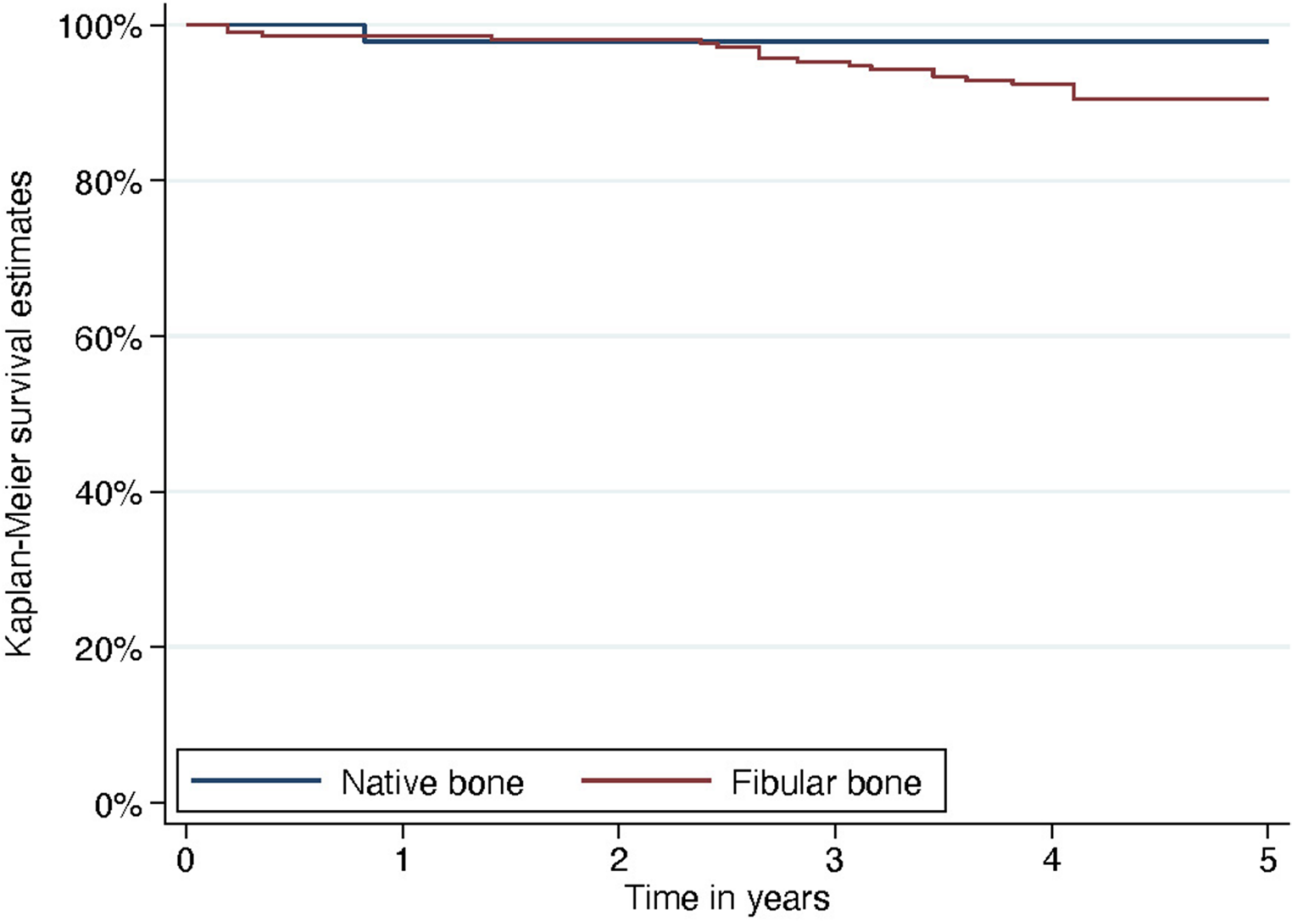
Kaplan-Meier survival analysis for dental implants in native bone (blue) and fibula bone (red)

A total of 53 patients (25 females and 28 males), with a mean age of 62.3 years (range: 15.6–85.4 years), were included in the study (Table 1). In total, 257 dental implants were placed, of which 210 were inserted into fibula grafts and 47 into native bone (mandible *n* = 9; maxilla *n* = 38). Within the observation period (mean observation period 64 months), three male patients with 16 dental implants (4 in the maxilla, 12 in the mandible) died. In this case, the last clinical evaluation of the patient was included, representing the end of the observation period. A total of 25 implants were lost during the follow-up period. Among the remaining 25 implant losses, 3 were classified as early failures and 22 as late failures. Of the 25 failed implants, 11 occurred in 5 patients who had received postoperative radiotherapy following FFF reconstruction. One implant failed in native bone, 2 implants failed in patients who had undergone radiotherapy prior to FFF, and 11 implants failed in patients without any history of irradiation. The cumulative implant survival rates were 98.4% at 12 months, 95.7% at 3 years, and 91.8% at 5 years (Fig. 2). Statistical analysis revealed no significant differences in survival between implants placed in fibula bone versus native bone (*p* = 0.122).

**Table 1:** Patients characteristics

Gender, n (%)	Female: 25 (47%)	
	Male: 28 (53%)	
Age in years, mean (range)	62.3 (15.6–85.4)	
Defect classification (n)	Mandible (HCL-classification):	H (4), L (18), C (4), LC (18), LCL (4)
	Maxilla (Brown classification):	Class I (1), Class II (4), Class III (0), Class IV (0), Class V (0), Class VI (0)
Number of segments, n (%)	1 Segment: 16 (30%*);* 2 Segments: 27 (51%) 3 Segments: 9 (17%) 4 Segmentes: 1 (2%)	
Radiation, n (%)	Prior to FFF reconstruction: 10 (19%) After reconstruction/adjuvant: 12 (23%) Non: 31 (58%)

### Occlusal status and prosthetic restoration

The occlusal status of the opposing arch varied, with the most common being partially edentulous (*n* = 19), followed by fully dentate (*n* = 13), partially edentulous with fixed prosthodontic restoration (*n* = 12), and implant-supported prostheses (*n* = 9). Most patients (*n* = 31) were rehabilitated with removable bar-supported overdentures. Fixed restorations were provided to 22 patients due to limited prosthodontic space and/or reduced mouth opening (*n* = 6) or partial jaw reconstruction (*n* = 16). The majority of reconstructions were performed using 2 fibula segments (*n* = 27), followed by 1 segment (*n* = 16), 3 segments (*n* = 9), and 4 segments (*n* = 1).

### Radiotherapy

Patients were stratified into 3 groups based on their radiotherapeutic status: (1) those who received surgical therapy alone, (2) those who received adjuvant radio(chemo)therapy following FFF reconstruction, and (3) those who underwent radio(chemo)therapy prior to FFF reconstruction. 

**Fig. 3:**
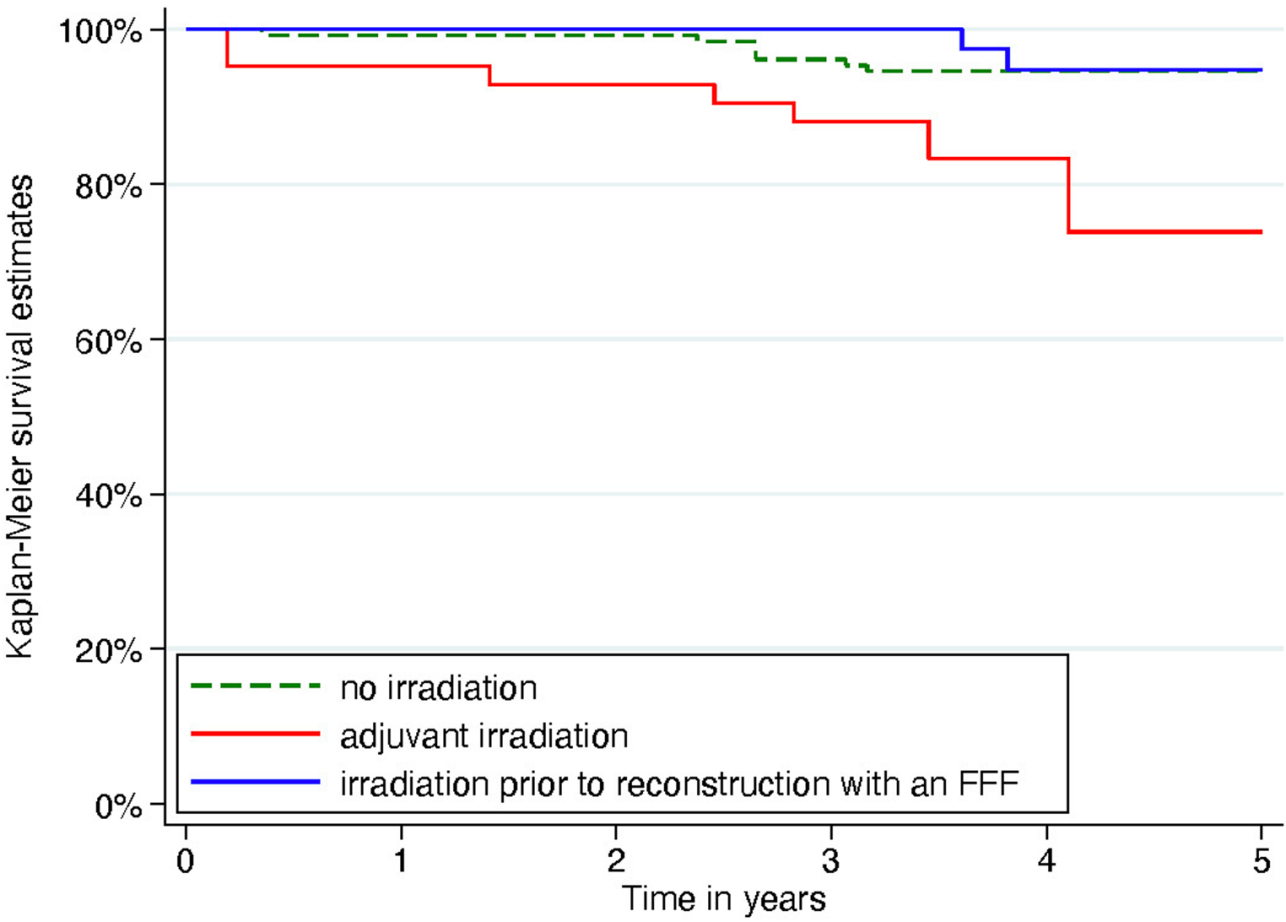
Kaplan-Meier survival analysis for dental implants in patients with no irradiation (green), adjuvant irradiation (red), and irradiation prior to reconstruction with an FFF (blue)

Of the 210 implants placed in fibula bone, 42 were inserted in patients with irradiation after FFF reconstruction, 39 in patients with irradiation prior to FFF, and 129 in non-irradiated patients. Among the 47 implants placed in native bone, 14 were in irradiated patients and 33 in non-irradiated patients Implant survival was assessed irrespective of implant site (native vs. fibula bone) and analyzed according to the corresponding group (Fig. 3):

1. Surgical treatment only: In 31 patients, 162 implants were placed without any history of radiotherapy. The cumulative survival rates were 99.2% (95% CI: 94.6%, 99.9%) at 1 year, 96.1% (95% CI: 90.9%, 98.4%) at 3 years, and 94.6% (95% CI: 89.0%, 97.4%) at 5 years.
2. Adjuvant irradiation after FFF reconstruction: In 12 patients, 42 implants were placed. The cumulative survival rate was 95.2% (95% CI: 82.3%, 98.8%) at 1 year, 88.1% (95% CI: 73.7%, 94.9%) at 3 years, and 73.8% (95% CI: 57.7%, 84.6%) at 5 years. This group exhibited the lowest survival rates with no significant differences between the groups (*p* = 0.310).
3. Irradiation prior to FFF reconstruction: Ten patients received radiotherapy before reconstruction, such that the FFF was not directly irradiated. Among these, 39 implants were placed, with only 2 failures during follow-up. Survival rates were 100% at 1 and 3 years and 94.8% (95% CI: 80.8%, 98.7%) at 5 years.

### Gender

Of the 257 total implants, 129 were placed in males and 128 in females. A total of 25 implant failures were recorded: 14 in females (2 early, 12 late) and 11 in males (3 early, 8 late). One year after placement, implant survival was 98.4% (95% CI: 93.9%, 99.6%) in male patients and 98.5% (95% CI: 93.9%, 99.6%) in female patients. At 3 years, survival declined to 96.9% (95% CI: 91.9%, 98.8%) in males and 94.6% (95% CI: 89.0%, 97.4%) in females. At 5 years, survival was 92.9% (95% CI: 86.9%, 96.3%) in males and 90.7% (95% CI: 84.2%, 94.6%) in females. No statistically significant difference in implant survival was observed between genders (*p* = 0.226).

### Jaw

In terms of anatomical distribution, 59 implants were inserted in the maxilla (21 in fibula bone, 38 in native bone) and 198 in the mandible (189 in fibula bone, 9 in native bone). Among the 25 failed implants, 24 were located in the fibula bone (after mandibular reconstruction), and 1 was placed in the native maxillary bone. Implant survival in the maxilla remained stable at 98.3% (95% CI: 88.6%, 99.8%) across 1, 3, and 5 years. In the mandible, survival was 98.5% (95% CI: 95.4%, 99.5%) at 1 year, decreasing to 95.0% (95% CI: 90.8%, 97.3%) at 3 years and 89.9% (95% CI: 84.8%, 93.4%) at 5 years. A significant difference (HR 4.8; *p =* 0.033) was found when adjusting for age, gender fibula status, implant diameter and implant length.

### Peri-implant bone level changes

**Fig. 4:**
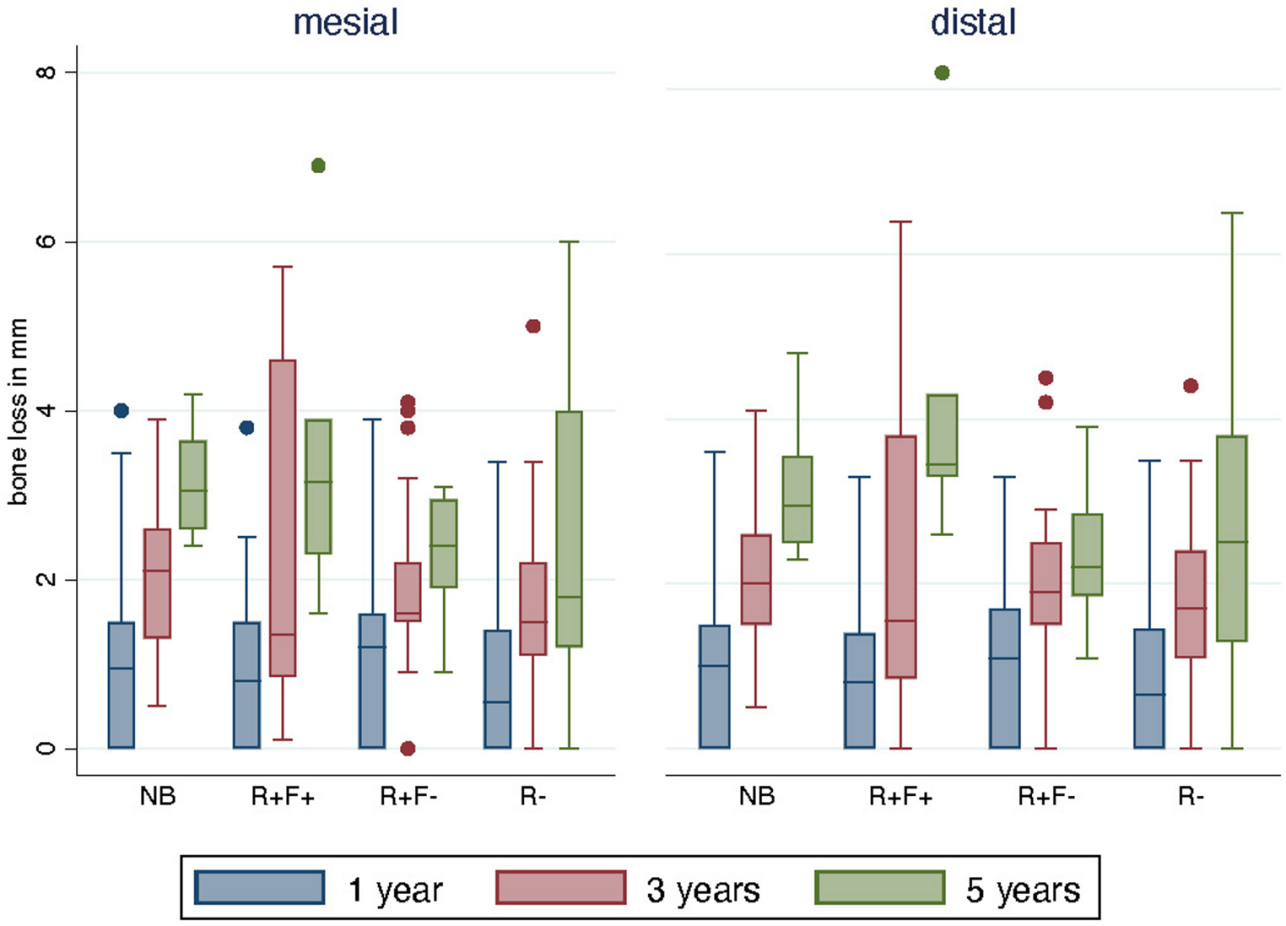
Mesial and distal bone level changes after 1 year (blue); 3 years (red), and 5 years (green) between native bone (NB), adjuvant radio(chemo)therapy after FFF (R + F+), patients who underwent radio(chemo)therapy prior to FFF reconstruction (R + F-), and patients with no adjuvant therapy (R-)

Early comparison of mesial and distal marginal bone loss revealed no statistically significant differences among the groups of implants placed in patients receiving adjuvant radio(chemo)therapy after FFF reconstruction, patients who underwent radio(chemo)therapy prior to FFF, and patients treated with surgery alone. This pattern was consistent at 1, 3, and 5 years (Fig. 4), except for a significant increase in distal bone loss at 5 years in the adjuvant radio(chemo)therapy after FFF reconstruction group (*p =* 0.009). The corresponding p-values were as follows: 1 year – mesial (*p =* 0.899), distal (*p =* 0.478); 3 years – mesial (*p =* 0.078), distal (*p =* 0.186); 5 years – mesial (*p =* 0.066).

**Fig. 5:**
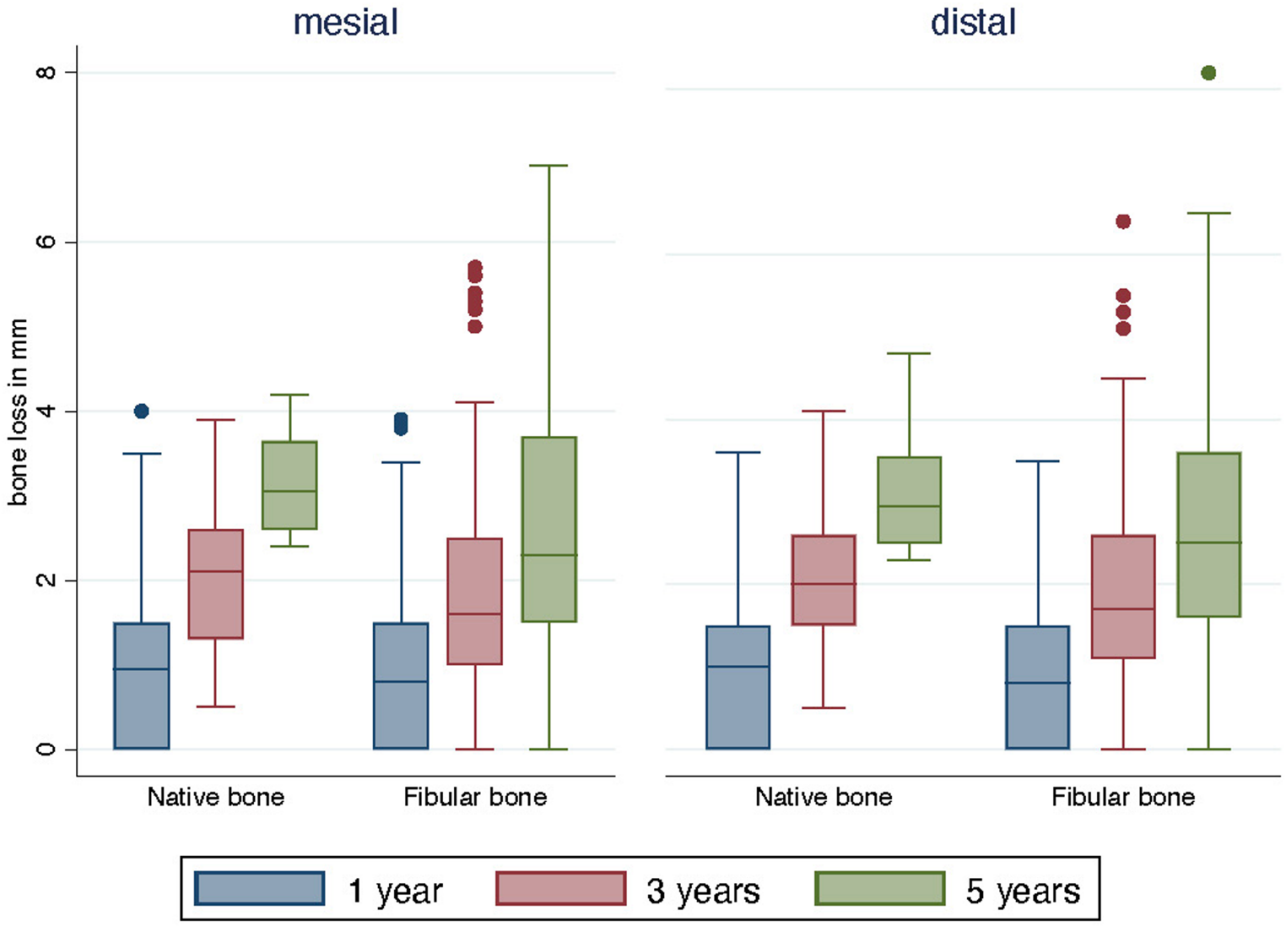
Mesial and distal bone level changes between native bone and fibula bone after 1 year (blue), 3 years (red), and 5 years (green)

When comparing marginal bone loss between fibula and native bone, slightly higher values were consistently observed in native bone, reaching statistical significance at 5 years for both mesial (*p =* 0.029) and distal sites (*p =* 0.039) (Fig. 5). Mean bone level changes with standard deviations for each group and site are summarized in Table 2.

**Table 2:** Mean mesial and distal peri-implant bone loss after 1, 3, and 5 years in millimeters

	1 year				3 years				5 years			
	Mesial		Distal		Mesial		Distal		Mesial		Distal	
	Mean	± SD	Mean	± SD	Mean	± SD	Mean	± SD	Mean	± SD	Mean	± SD
Overall	**0.9**	0.9	**0.9**	0.9	**1.9**	1.2	**1.9**	1.2	**2.7**	1.4	**2.9**	1.5
NB	**1.1**	1.0	**1.2**	1.1	**2.0**	0.9	**2.0**	1.0	**3.2**	0.6	**3.2**	0.8
FB	**0.8**	0.9	**0.9**	0.8	**1.8**	1.3	**1.9**	1.3	**2.6**	1.6	**2.8**	1.7
R+ F+	**0.9**	0.9	**0.9**	0.8	**2.4**	2.0	**2.4**	1.9	**3.5**	1.9	**4.2**	2.0
R+ F-	**1.0**	0.9	**1.0**	0.8	**1.9**	1.0	**2.0**	1.1	**2.3**	0.7	**2.3**	0.8
R-	**0.8**	0.8	**0.8**	0.8	**1.6**	1.0	**1.7**	1.1	**2.4**	1.8	**2.7**	1.8

*NB*: native bone; *FB*: fibula bone; *R+ F+*: radiotherapy after FFF; *R+ F-*: radiotherapy prior to FFF; *R-*, non-irradiated patients.

### Gender

When analyzed by gender, mesial and distal bone loss was generally lower in female patients compared to males at 1 and 3 years. However, at the 5-year mark, female patients demonstrated slightly greater bone loss. Nevertheless, these differences were not statistically significant. Corresponding mean values and standard deviations are provided in Table 3.

**Table 3:** Mean mesial and distal peri-implant bone loss after 1, 3, and 5 years in millimeters

	1 year				3 years				5 years			
	Mesial		Distal		Mesial		Distal		Mesial		Distal	
	Mean	± SD	Mean	± SD	Mean	± SD	Mean	± SD	Mean	± SD	Mean	± SD
Male	**1.0**	1.0	**1.0**	0.8	**1.9**	1.3	**2.0**	1.3	**2.6**	1.3	**2.8**	1.6
Female	**0.8**	0.8	**1.0**	0.8	**1.8**	1.1	**1.9**	1.2	**3.0**	1.4	**3.2**	1.2

## Discussion

After tumor resection, primary or secondary osseous reconstruction of the jaw is often required [33] and generally marks the beginning of the reconstruction of orofacial form and function [29, 34]. Within this context, the microvascular FFF remains the most used bone graft for the reconstruction of the jaw, especially the mandible [35–38]. Consecutive implant-retained prosthodontic rehabilitation completes the restoration of orofacial form and function [29, 34]. This study aimed to provide insights into long-term dental implant survival as well as peri-implant bone loss in patients, with special attention given to radiotherapy and its effect on gender and jaw.

The overall cumulative dental implant survival rate within the present study was 98.4% at 1 year, 95.7% after 3 years, and 91.8% at 5 years. To date, there are only a few studies in the literature on long-term survival rates following FFF. Ritschl et al. reported a cumulative dental implant survival rate of 83.3% over a median surveillance period of 32 months (range: 15–56 months), which is lower than our findings at the 5-year mark [20]. In Ritschl et al.’s study, 60 dental implants were placed in 22 patients, and 8 implants were lost. The implants’ survival and success were scored based on the 2008 Pisa consensus in 4 different groups (success, satisfactory survival, compromised survival, and failure), which differs from the present study’s use of the Kaplan-Meier method. However, a total of 28 out of 52 (53.8%) dental implants were loaded and matched those success criteria after the observation period. The method used in the present study revealed the estimated percent survival of the implants at each time point without further clarification of success criteria. In consideration of these points, 50 out of 60 (83.3%) implants survived in Ritschl et al.’s study after 32 months. Sozzi et al. also published data from 22 patients after a mean observation period of 7.8 years, with a survival rate of 98%, but these patients were not only tumor patients [39]. Patients with extreme alveolar atrophy were also included, which limits the comparability of these data to the present study. Attia et al. investigated 34 patients with dental implants after tumor surgery and reconstruction with an FFF. Here, the 1- and 11-year cumulative survival rates were 93% and 78%. Nevertheless, these studies included fewer patients and/or fewer dental implants [20, 23, 39] in comparison to the present study. Moreover, inconsistencies were observed in the method of dental implant placement and the approach used to assess crestal bone loss [20, 40]. Another study by Ewers al. investigated the long-term success and survival of extra-short and short locking-taper dental implants in fibula grafts with follow-up periods of up to 14 years, placing particular emphasis on potential factors influencing outcomes. However, their study included fewer patients and implants and reported a shorter mean follow-up (54.2 months) [41]. These studies highlight the challenges in collecting and standardizing long-term data for patients following FFF reconstruction and dental implant placement, emphasizing the significance of the presented study.

Additionally, our study investigated dental implant survival in fibula bone and native bone. While native bone demonstrated higher dental implant survival rates at 3 and particularly 5 years, the difference was not significant. All lost implants except 1 within this study were located in fibula bone. Comparisons of dental implant survival between native and fibula bone remain inconsistent in the literature and are rarely studied. It has already been demonstrated that alveolar bone and fibula bone differ significantly in terms of some bone morphological parameters, such as osteocyte lacunar density [42]; however, this distinction does not apply, for instance, to osteoblast morphology [43]. In this context, it is important to note that most patients in this study received osseous reconstructions using either one or two fibula segments. Subsequently, most patients were rehabilitated with a removable bar-supported overdenture, combining native and graft bone. Due to the stabilizing function of the bar, it can be assumed that high stabilization between the different bone entities can be achieved, which might have a positive influence on dental implant survival. Although the current literature does not provide conclusive evidence favoring a specific attachment system [44], a randomized controlled trial demonstrated that 4-implant bar-retained overdentures exhibited the highest retention, which is a particularly important criterion for our specific patient population [45]. Sozzi et al. (2017) also reported no statistical differences between implants placed in native versus fibula bone. However, their study included only 8 dental implants placed in native bone [39]. In contrast, Flores-Ruiz et al. (2018) observed significantly higher dental implant survival in native bone (90.1%) compared to graft bone (73.3%). Although their study provides data up to 5 years, it does not include specific details about the bone grafts used [46]. In summary, our findings suggest that a removable bar-supported overdenture with dental implants in both native and fibula bone achieves additional stability and therefore provides a reliable prosthetic solution for bony reconstructions while simultaneously facilitating improved oral hygiene.

The influence of radiation/radiochemotherapy on long-term implant survival is also described contradictorily in the literature [28, 47, 48]. Furthermore, long-term data remain limited in cases involving augmented bone. Schiegnitz et al. published representative data of 711 implants in 164 oral cancer patients. Implants inserted in irradiated bone that received augmentation procedures showed statistically significantly lower implant survival. Without a specific subdivision into FFF and iliac bone grafts, the 5-year data survival rate was lower than 50% [49]. In the presented long-term study, the comparison went beyond irradiated and non-irradiated patients. Thus, 3 groups were formed to distinguish between patients who received adjuvant radio(chemo)therapy, radio(chemo)therapy prior to FFF reconstruction, and non-irradiated patients. Within these groups, the cumulative survival rate for dental implants was found to be remarkedly lower after 5 years in patients with adjuvant radiation (73.8%) when compared to patients who received radiotherapy to the head and neck region at an earlier stage (94.8%) and patients with merely surgical therapy (94.6%). Of the reported 25 lost implants, 14 were located in irradiated bone. These findings fall within the range of results reported by Lodders et al., who documented long-term dental implant survival rates of 55.3% in irradiated FFFs and 96% in non-irradiated FFFs [24]. Investigated the survival of the implants after reconstruction with an FFF in oncological patients and revealed a 5-year survival rate of 83.1%. In this study, only 37 of 108 implants were irradiated and further investigated [35]. All in all, it appears that the factor of radiation has a considerable impact on the survival rate of implants and should be given particular consideration.

Nevertheless, it must be acknowledged that in our study, the non-irradiated patients were not further stratified according to their smoking status. Continued smoking was documented in 4 patients, all of whom lost 5 dental implants (all placed in the transplanted fibula bone). This aspect has been investigated in only a few studies, such as the work by Lodders et al. [24]. In most of the cited studies, the reporting on smoking status is either inconsistent or unclear. For example, past and current smoking status was not collected by Ritschl et al. [20] or was not explicitly mentioned [23, 35, 47]. A recently published study demonstrated that postoperative radiotherapy in osseous free flaps is associated with a higher rate of complications and the need for secondary surgeries [50]. Furthermore, the applied mean dose (Dmean), maximum dose (Dmax), and volumes exposed to 35–70 Gy (V35-70) were calculated from the dose-volume histograms in 5 Gy increments [50], which would have been of particular interest in the present study. However, this was not feasible due to the retrospective nature of our analysis, and it should be addressed in future prospective studies. The authors of that study also raised the question of whether applying high radiation doses to the bone flap with its vulnerable tissue may contribute to adverse outcomes [50], a concern that is further supported by our results regarding dental implant survival in such patients.

Additionally, for patients undergoing ablative surgery and potentially adjuvant radio(chemo)therapy, maintaining healthy peri-implant tissues may play a crucial role in implant success. In this context, previous studies [51–53] have highlighted the importance of keratinized gingiva in peri-implant tissue health [54] and peri-implant bone level. Moreover, a proteomic analysis revealed that de novo regenerated mucosa overlying the FFF adopts active tissue function, closely resembling the characteristics of oral keratinized mucosa [55], indicating the regenerative potential of the FFF. The implant survival rate is a coarse, binary outcome (yes/no), while bone level changes offer a quantitative, nuanced, and clinically meaningful understanding of implant performance.

Within the present study, mean bone level changes of 0.9 mm (mesial and distal) after 1 year, 1.9 mm (mesial and distal) after 3 years, and 2.7 mm mesial and 2.9 mm distal after 5 years were reported. Regarding marginal bone resorption in patients after reconstruction with FFF and dental implant placement, there is still little data. Wang et al. examined the peri-implant bone resorption in patients after mandibular reconstruction with double-barrel fibula (DBF) bone grafting or vertical distraction osteogenesis fibula (VDOF). After 3 years, mean peri-implant bone resorption was found to be 0.68 mm for DBF and 0.71 mm for VDOF [56]. Nevertheless, it must be mentioned that Wang et al. only included patients with benign tumors of the mandible (keratocysts and ameloblastoma) who did not undergo radiotherapy and were younger than the presented study’s patients (43.4 years), and they carried out a shorter follow-up period (42.5 months) [56]. In 2021, Li et al. investigated the clinical and radiological parameters of 19 patients after FFF or iliac flap in tumor and trauma patients. Here, bone level changes of 0.6 mm were documented after a 26-month observation period, but with an incidence of peri-implantitis of 32.2%. Consequently, the long-term outcome is difficult to predict [57]. In a previous study, the peri-implant bone loss of dental implants inserted in fibula bone after 5 years was similar to that observed in this study (mesial 2.3 vs. 2.6 mm; distal 2.3 vs. 2.8 mm), but the total number of patients included was smaller, and potentially influencing parameters (such as radiation) were not differentiated [19]. Duttenhoefer et al. examined peri-implant bone loss in non-vascularized fibula bone flaps in patients suffering from Class VI atrophy and less than 5 mm residual bone volume, showing a mean bone resorption after 10 years of 1.4 mm [58]. In this study, no patients with oral cancer and/or irradiation were included. Therefore, it can be assumed that the FFF has a completely different healing environment, and the soft tissue conditions also differ. So, the results are only comparable to a limited extent.

In the present study, a distinction regarding the bone level changes was made for the first time between irradiation and non-irradiation of fibula bone or local bone. While the peri-implant bone resorption rates were comparable in the group without irradiation and the group with previous irradiation but non-irradiated fibula bone, the distal resorption rates were significantly higher in the group with adjuvant radio(chemo)therapy after 5 years, which might contribute to the lower dental implant survival after 5 years in this group. The extent to which additional parameters, such as localization and the localization-related dose, play a role would need to be clarified in further studies. In case of irradiated fibula bone, the surrounding soft tissue appears to be even more complex and needs more attention, even in follow-up protocols and hygiene measures. Additionally, since the bone level changes in fibula bone were significantly lower after 5 years (mesial and distal) when compared to native bone, it may be concluded that soft tissue management with a vestibuloplasty generates long-term stable peri-implant conditions, also in vulnerable situations.

Overall, it is important to emphasize that the documented rates of bone resorption should be regarded as favorable, particularly when compared to established success criteria, such as those defined by Albrektsson [59]. One must bear in mind that the reconstructed site does not consist of native (mandibular/maxillary) bone and that the surrounding soft tissue conditions differ significantly. Nevertheless, the prosthetic rehabilitation of such highly complex cases is feasible and continues to improve—particularly through more precise preoperative planning of bone segment positioning and implant-retained prosthetic rehabilitation.

## Conclusion

Irradiation reduces the survival of implants in FFF as a decisive parameter. Implant survival is higher in non-irradiated patients or patients who received irradiation prior to FFF reconstruction. Moreover, an approximately 1.5-fold increase in crestal bone level changes was observed in implants placed in irradiated FFF after five years.

### Limitation

The inclusion of a diverse patient population with varying systemic and local risk factors may have influenced the outcomes and introduced variability. Particularly, the inclusion of 4 smokers in the non-irradiated group should be explicitly mentioned in this context. Likewise, the lack of correlation between the applied mean (Dmean) and maximum doses (Dmax) in irradiated patients should be recognized as a limitation of the study. Reliance solely on radiographic bone level measurements without clinical parameters (e.g. probing depth) may limit the comprehensive assessment of peri-implant health. In this context, the absence of peri-implant clinical parameters, such as bleeding on probing and the incidence of peri-implant mucositis or peri-implantitis, is certainly of importance, while their omission in this study also represents a limitation.

## Supplementary Information

Below is the link to the electronic supplementary material.

Supplementary Material 1

## Data Availability

The participants of this study did not give written consent for their data to be shared publicly, so due to the sensitive nature of the research supporting data is not available.
